# Supplementary material for: Prevalence of Asthma and Its Associating Environmental Factors among 6–12-Year-Old Schoolchildren in a Metropolitan Environment—A Cross-Sectional, Questionnaire-Based Study
Source: Int J Environ Res Public Health. 2021 Dec 20;18(24):13403. doi: 10.3390/ijerph182413403 (PMC8709131; doi:10.3390/ijerph182413403)
Supplement: Supplementary file 1 [file ijerph-18-13403-s001.zip › Table_S1.pdf]

**Table S1:** Dataset of environmental risk factor analysis

| QID | Environmental factor | | n (%) in CA | p-values | OR | CI |
|---|---|---|---|---|---|---|
| E1 | Location of residence in the suburbs (according to ZIPcodes) | Yes (n= 346) | 30 (8.67) | 0.0236 | 0.64 | 0.43 – 0.93 |
| | | No (n= 3464) | 448 (12.93) | | | |
| E2 | Living in a house with prefabricated concrete walls | Yes (n= 1013) | 135 (13.33) | 0.4282 | 1.09 | 0.88 - 1.35 |
| | | No (n= 2823) | 349 (12.36) | | | |
| E3 | Living in a house with brick walls | Yes (n= 1579) | 191 (12.10) | 0.4165 | 0.92 | 0.76 - 1.12 |
| | | No (n= 2257) | 293 (12.98) | | | |
| E4 | Living in a detached house surrounded by a garden | Yes (n= 1570) | 191 (12.17) | 0.4833 | 0.93 | 0.77 - 1.13 |
| | | No (n= 2266) | 293 (12.93) | | | |
| E5 | Living in a log cabin | Yes (n= 6) | 2 (33.33) | 0.1514 | 3.47 | 0.48 - 17.85 |
| | | No (n= 3830) | 482 (12.58) | | | |
| E6 | Wall-to-wall carpet in the bedroom | Yes (n= 611) | 88 (14.40) | 0.1479 | 1.20 | 0.93 - 1.53 |
| | | No (n= 3225) | 396 (12.28) | | | |
| E7 | Linoleum in the bedroom | Yes (n= 76) | 13 (17.11) | 0.2367 | 1.44 | 0.75 - 2.55 |
| | | No (n= 3760) | 471 (12.53) | | | |
| E8 | Parquet in the bedroom | Yes (n= 3374) | 426 (12.63) | 0.9652 | 1.01 | 0.76 - 1.36 |
| | | No (n= 462) | 58 (12.55) | | | |
| E9 | Tapestry in the bedroom | Yes (n= 183) | 24 (13.11) | 0.8355 | 1.05 | 0.66 - 1.60 |
| | | No (n= 3653) | 460 (12.59) | | | |
| E10 | Visible mould in the bedroom | Yes (n= 122) | 28 (22.95) | 0.0006 | 2.13 | 1.36 – 3.24 |
| | | No (n= 3714) | 456 (12.28) | | | |
| E11 | Plant in the bedroom | Yes (n= 1543) | 171 (11.08) | 0.0191 | 0.79 | 0.65 – 0.96 |
| | | No (n= 2293) | 313 (13.65) | | | |
| E12 | Feather bedding when sleeping | Yes (n= 546) | 64 (11.72) | 0.4964 | 0.91 | 0.68 – 1.19 |
| | | No (n= 3290) | 420 (12.77) | | | |
| E13 | Heating with wood or coal burning stove | Yes (n= 97) | 8 (8.25) | 0.1935 | 0.62 | 0.27 - 1.20 |
| | | No (n= 3739) | 476 (12.73) | | | |
| E14 | Heating with fuel oil stove | Yes (n= 17) | 3 (17.65) | 0.5342 | 1.49 | 0.34 - 4.58 |
| | | No (n= 3819) | 481 (12.59) | | | |
| E15 | Central heating | Yes (n= 3274) | 418 (12.77) | 0.4999 | 1.10 | 0.84 - 1.46 |
| | | No (n= 562) | 66 (11.74) | | | |

| | | | | | | |
|---|---|---|---|---|---|---|
| E16 | Heating with wood or coal burning stove during the first year of the child's life | Yes (n= 120) | 20 (16.67) | 0.1768 | 1.40 | 0.84 - 2.24 |
| | | No (n= 3716) | 464 (12.49) | | | |
| E17 | Heating with fuel oil stove during the first year of the child's life | Yes (n= 16) | 2 (12.50) | 0.9887 | 0.99 | 0.15 - 3.56 |
| | | No (n= 3820) | 482 (12.62) | | | |
| E18 | Central heating during the first year of the child's life | Yes (n= 3282) | 414 (12.61) | 0.9890 | 0.10 | 0.77 - 1.32 |
| | | No (n= 554) | 70 (12.64) | | | |
| E19 | Frequent use of an air conditioner (1 – 2 hours / day) | Yes (n= 854) | 105 (12.30) | 0.7478 | 0.96 | 0.76 - 1.21 |
| | | No (n= 2982) | 379 (12.71) | | | |
| E20 | Heavy-vehicle traffic frequent or constant (in 500 m) | Yes (n= 2483) | 337 (13.57) | 0.0161 | 1.29 | 1.05- 1.59 |
| | | No (n= 1353) | 147 (10.86) | | | |
| E21 | Living in a weedy area (in 500 m) | Yes (n= 1666) | 245 (14.71) | 0.0007 | 1.39 | 1.15 - 1.69 |
| | | No (n= 2170) | 239 (11.01) | | | |
| E22 | Living not far from an air-polluting factory or mine (in 1 km) | Yes (n= 689) | 106 (15.38) | 0.0161 | 1.33 | 1.05 - 1.68 |
| | | No (n= 3147) | 378 (12.01) | | | |
| E23 | Keeping furry animals or birds at home | Yes (n= 1373) | 178 (12.96) | 0.6291 | 1.05 | 0.86 – 1.28 |
| | | No (n= 2463) | 306 (12.42) | | | |
| E24 | Dogs at home | Yes (n= 621) | 101 (16.26) | 0.0029 | 1.44 | 1.13 – 1.82 |
| | | No (n= 3215) | 383 (11.91) | | | |
| E25 | Cats at home | Yes (n= 416) | 48 (11.54) | 0.4831 | 0.89 | 0.64 – 1.21 |
| | | No (n= 3420) | 436 (12.75) | | | |
| E26 | Rodents at home | Yes (n= 481) | 47 (9.77) | 0.0454 | 0.72 | 0.52 – 0.98 |
| | | No (n= 3355) | 437 (13.03) | | | |
| E27 | Birds at home | Yes (n= 111) | 15 (13.51) | 0.7730 | 1.08 | 0.60 – 1.83 |
| | | No (n= 3725) | 469 (12.59) | | | |
| E28 | Furry animals or birds at home in the first year of the child's life | Yes (n= 683) | 94 (13.76) | 0.3204 | 1.13 | 0.88 – 1.43 |
| | | No (n= 3153) | 390 (12.37) | | | |
| E29 | Dogs at home in the first year of the child's life | Yes (n= 429) | 61 (14.22) | 0.2897 | 1.17 | 0.87 – 1.55 |
| | | No (n= 3407) | 423 (12.42) | | | |
| E30 | Cats at home in the first year of the child's life | Yes (n= 240) | 33 (13.75) | 0.5855 | 1.11 | 0.75 - 1.60 |
| | | No (n= 3596) | 451 (12.54) | | | |
| E31 | Rodents at home in the first year of the child's life | Yes (n= 117) | 15 (12.82) | 0.9464 | 1.02 | 0.56 – 1.71 |
| | | No (n= 3719) | 469 (12.61) | | | |

| | | | | | | |
|---|---|---|---|---|---|---|
| E32 | Birds at home in the first year of the child's life | Yes (n= 42) | 9 (21.43) | 0.0892 | 1.91 | 0.85 – 3.84 |
| | | No (n= 3794) | 475 (12.52) | | | |
| E33 | Smoking at home in the first year of the child's life | Yes (n= 325) | 59 (18.15) | 0.0018 | 1.61 | 1.18 – 2.16 |
| | | No (n= 3511) | 425 (12.10) | | | |
| E34 | Smoking at home | Yes (n= 370) | 68 (18.38) | 0.0005 | 1.65 | 1.24 – 2.18 |
| | | No (n= 3466) | 416 (12.00) | | | |

QID: question ID, CA: cumulative asthma, OR: odds ratio, CI: confidence interval.
