# Supplementary material for: Prevalence of Asthma and Its Associating Environmental Factors among 6–12-Year-Old Schoolchildren in a Metropolitan Environment—A Cross-Sectional, Questionnaire-Based Study
Source: Int J Environ Res Public Health. 2021 Dec 20;18(24):13403. doi: 10.3390/ijerph182413403 (PMC8709131; doi:10.3390/ijerph182413403)
Supplement: Supplementary file 1 [file ijerph-18-13403-s001.zip › Table_S2.pdf]

Table S2: Dataset of nutritional risk factor analysis

QID	Nutritional factors: Weekly frequency of ingredient consumption during the last 12 months		n (%) in CA	p-values	OR	CI
N1d	Meat	Frequently [≥1 times] (n= 3772)	474 (12.57)	0.7988	1.11	0.55 - 2.69
		Rarely [<1 time] (n= 61)	7 (11.48)			
		None responders (n= 3)	3 (100.00)			
N2d	Fish or seafood	Frequently [≥1 times] (n= 1583)	209 (13.20)	0.3603	1.09	0.90 - 1.33
		Rarely [≤2 times] (n= 2253)	275 (12.21)			
N3d	Fresh fruit	Frequently [≥1 time] (n= 3696)	467 (12.64)	0.8633	1.05	0.64 - 1.82
		Rarely [<1 time] (n= 140)	17 (12.14)			
N4d	Fresh vegetables	Frequently [≥1 time] (n= 3489)	437 (12.53)	0.5856	0.91	0.67 - 1.28
		Rarely [<1 time] (n= 347)	47 (13.54)			
N5d	Legumes (e.g. bean, pea, lentils)	Frequently [≥1 time] (n= 2681)	338 (12.61)	0.9772	0.10	0.81 - 1.23
		Rarely [<1 time] (n= 1155)	146 (12.64)			
N6d	Cereals (e.g. wheat, rye)	Frequently [≥1 time] (n= 3517)	420 (11.94)	0.0000	0.54	0.41 - 0.73
		Rarely [<1 time] (n= 319)	64 (20.06)			
N7d	Pasta	Frequently [≥1 time] (n= 3722)	475 (12.76)	0.1275	1.71	0.91 - 3.65
		Rarely [<1 time] (n= 114)	9 (7.89)			
N8d	Rice	Frequently [≥1 time] (n= 3539)	450 (12.72)	0.5278	1.13	0.79 - 1.66
		Rarely [<1 time] (n= 297)	34 (11.45)			
N9d	Butter	Frequently [≥1 time] (n= 3053)	393 (12.87)	0.3475	1.12	0.89 - 1.44
		Rarely [<1 time] (n= 783)	91 (11.62)			
N10d	Margarine	Frequently [≥1 time] (n= 2214)	310 (14.00)	0.0026	1.35	1.11 - 1.65
		Rarely [<1 time] (n= 1622)	174 (10.73)			
N11d	Nuts (e.g. chestnut, peanut, almond)	Frequently [≥1 time] (n= 2088)	247 (11.83)	0.1087	0.86	0.71 - 1.04
		Rarely [<1 time] (n= 1748)	237 (13.56)			

QID	Nutritional factors: Weekly frequency of ingredient consumption during the last 12 months		n (%) in CA	p-values	OR	CI
N12d	Potatoe	Frequently [≥1 time] (n= 3584)	456 (12.72)	0.4569	1.17	0.79 - 1.78
		Rarely [<1 time] (n= 252)	28 (11.11)			
N13d	Milk or dairy products	Frequently [≥1 time] (n= 3328)	417 (12.53)	0.6771	0.94	0.72 - 1.25
		Rarely [<1 time] (n= 508)	67 (13.19)			
N14d	Egg	Frequently [≥1 time] (n= 3324)	415 (12.48)	0.5296	0.92	0.70 - 1.21
		Rarely [<1 time] (n= 512)	69 (13.48)			
N15d	Fast food (e.g. hamburger)	Frequently [≥1 time] (n= 622)	115 (18.49)	0.0000	1.75	1.39 - 2.19
		Rarely [<1 time] (n= 3214)	369 (11.48)			
N16d	Soft drinks or juices containing colorants or preservatives	Frequently [≥1 time] (n= 1550)	220 (14.19)	0.0157	1.27	1.05 - 1.53
		Rarely [<1 time] (n= 2286)	264 (11.55)			

QID: question ID, CA: cumulative asthma, OR: odds ratio, CI: confidence interval.
