# Supplementary material for: Prevalence of Asthma and Its Associating Environmental Factors among 6–12-Year-Old Schoolchildren in a Metropolitan Environment—A Cross-Sectional, Questionnaire-Based Study
Source: Int J Environ Res Public Health. 2021 Dec 20;18(24):13403. doi: 10.3390/ijerph182413403 (PMC8709131; doi:10.3390/ijerph182413403)
Supplement: Supplementary file 1 [file ijerph-18-13403-s001.zip › Table_S3.pdf]

Table S3: Dataset of cumulative asthma prevalence in BMI-for-age percentile ranges

**BMI-for-age percentile**	**00 - 03**	**03 - 10**	**10 - 75**	**75 - 90**	**90 - 97**	**97 – 100**
Cumulative asthma (n= 401)	28 (9.40%)	40 (11.08%)	212 (11.53%)	68 (13.77%)	32 (15.09%)	21 (23.08%)
Non-asthmatic (n= 2894)	270	321	1627	426	180	70

Chi-square= 16.26 df= 5 p= 0.0062
